# 

**Published:** 1844-02

**Authors:** 


### WESTERN JOURNAL

OF

### MEDICINE AND SURGERY.

### EDITORS.

---

DANIEL DRAKE, M. D.,

PROFESSOR OF PATHOLOGICAL ANATOMY AND CLINICAL MEDICINE IN THE MEDICAL INSTITUTE OF LOUISVILLE:

LUNSFORD P. YANDELL, M. D.,

PROFESSOR OF CHEMISTRY AND PHARMACY IN THE SAME:

THOMAS W. COLESCOTT, M. D.

### TO CORRESPONDENTS, &c.

Dr. Drake expects to commence a second tour through the States of Louisiana, Florida, Mississippi and Alabama, early in the spring, in the prosecution of his design of investigating the diseases of the south-west in the localities where they appear. He will receive and forward communications for the Journal. Drs. Yandell and Colescott will remain in Louisville during the summer, where they may be addressed by their correspondents. All business communications to be addressed to the publishers, Messrs. Prentice & Weissinger. Y.

---

### WESTERN JOURNAL.

The delay incident to the procuring of new type is the cause of the late appearance of this and the previous number of our Journal, but our March number will be issued in better time, and thereafter we expect to be able to get the work out punctually at the beginning of the month. We are resolved on setting our subscribers a worthy example of punctuality. Let them emulate it, and the Journal will be placed beyond the reach of any contingency.

### RECEIPTS FOR THE MEDICAL JOURNAL.

| | |
|---|---|
| Dr. S. Jackson, Columbus, Ia., - - - - | $2 00 |
| F. Chapellin, Natchez, Miss., - - - - | 5 00 |
| D. W. Strader, Centre P. O. Ky. - - - | 13 75 |
| P. Winfree, New River, La. - - - - | 5 00 |
| T. Booth, Clarksville, Mo., - - - - | 10 00 |
| J. D. Mason, Jackson, Tenn., - - - - | 5 00 |
| New-York Medical Lyceum, New-York, N. Y. - - | 5 00 |
| J. N. Dorsey, Owensboro, Ky. - - - - | 5 00 |
| W. M. Dodson, Erie, Mo. - - - - | 5 00 |
| Dr. J. W. Beauchamp, Edmonton, Ky., - - - | 5 00 |
| D. Dayton, South Bend, Ia., - - - - | 5 00 |
| Dr. W. F. Brown, Spring Hill, Tenn., - - - | 10 00 |
| W. A. Manny, White Hall, Tenn. - - - | 5 00 |
| Dr. Jas. Clayton, Christiansburg, Ky., - - - | 5 00 |
| J. H. Dunlery, Beatty's Bluff, Miss., - - - | 5 00 |
| J. P. Smith, Starkville, Miss., - - - - | 5 00 |

### TO OUR SUBSCRIBERS.

We must again remind our subscribers of their arrearages to us, and *urge* them to remit their respective dues. Our expenses for the last and present volume have been more than usually heavy, whilst the receipts, we are sorry to say, have by no means augmented in like proportion. Those who owe for this Journal from the commencement, will not, we trust, suffer us to ask them again for the amounts due us. We hope, moreover, that *all* our subscribers, whether old or recent, will feel it incumbent to remit immediately.

Thus far, we have not collected enough to defray the cost of the Journal, but we are determined to sustain it. We design commencing the ninth volume, with new and improved materials—will not the subscribers encourage and sustain us by remitting forthwith by mail, at our risk, franked by the Postmaster?

*October*, 1843. PRENTICE & WEISSINGER.

The Western Journal of Medicine and Surgery is published monthly by the undersigned, at the corner of Main and Fifth streets, Louisville, at $5 per annum, payable in advance. Each number contains 96 pages, making two volumes in the year of more than 550 pages.

Contributions to its pages by the Physicians of the Valley of the Mississippi, addressed to the Editors, are respectfully solicited.

Letters on business, to be addressed, postage paid, to the publishers.

☞Postmasters, by the regulations of the Postoffice Department, will frank letters containing subscription money, and all remittances so franked are at the risk of the publishers.

*January*, 1844. PRENTICE & WEISSINGER.

